# Investigation of chronic and persistent classical swine fever infections under field conditions and their impact on vaccine efficacy

**DOI:** 10.1186/s12917-019-1982-x

**Published:** 2019-07-15

**Authors:** Liani Coronado, Jose Alejandro Bohórquez, Sara Muñoz-González, Lester Josue Perez, Rosa Rosell, Osvaldo Fonseca, Laiyen Delgado, Carmen Laura Perera, Maria Teresa Frías, Llilianne Ganges

**Affiliations:** 1 National Centre for Animal and Plant Health (CENSA), OIE Collaborating Centre for Disaster Risk Reduction in Animal Health, San José de las Lajas, Mayabeque Cuba; 2 OIE Reference Laboratory for Classical Swine Fever, IRTA-CReSA, Campus de la Universitat Autònoma de Barcelona, 08193 Bellaterra, Spain; 3 University of Illinois, College of Veterinary Science, Department of Clinical Veterinary Medicine, Urbana, Illinois 61802 United States; 4 Departament d’Agricultura Ramaderia i Pesca (DARP), Centre de Recerca en Sanitat Animal (CReSA, IRTA-UAB), Campus de la Universitat Autònoma de Barcelona, 08193 Bellaterra, Spain

**Keywords:** CSFV, Chronic infection, Persistent infection, Vaccination failures, Viral evolution

## Abstract

**Background:**

Recent studies have hypothesized that circulation of classical swine fever virus (CSFV) variants when the immunity induced by the vaccine is not sterilizing might favour viral persistence. Likewise, in addition to congenital viral persistence, CSFV has also been proven to generate postnatal viral persistence. Under experimental conditions, postnatal persistently infected pigs were unable to elicit a specific immune response to a CSFV live attenuated vaccine via the mechanism known as superinfection exclusion (SIE). Here, we study whether subclinical forms of classical swine fever (CSF) may be present in a conventional farm in an endemic country and evaluate vaccine efficacy under these types of infections in field conditions.

**Results:**

Six litters born from CSF-vaccinated gilts were randomly chosen from a commercial Cuban farm at 33 days of age (weaning). At this time, the piglets were vaccinated with a lapinized live attenuated CSFV C-strain vaccine. Virological and immunological analyses were performed before and after vaccination. The piglets were clinically healthy at weaning; however, 82% were viraemic, and the rectal swabs in most of the remaining 18% were positive. Only five piglets from one litter showed a specific antibody response. The tonsils and rectal swabs of five sows were CSFV positive, and only one of the sows showed an antibody response. After vaccination, 98% of the piglets were unable to clear the virus and to seroconvert, and some of the piglets showed polyarthritis and wasting after 36 days post vaccination. The CSFV E2 glycoprotein sequences recovered from one pig per litter were the same. The amino acid positions 72(R), 20(L) and 195(N) of E2 were identified in silico as positions associated with adaptive advantage.

**Conclusions:**

Circulation of chronic and persistent CSF infections was demonstrated in field conditions under a vaccination programme. Persistent infection was predominant. Here, we provide evidence that, in field conditions, subclinical infections are not detected by clinical diagnosis and, despite being infected with CSFV, the animals are vaccinated, rather than diagnosed and eliminated. These animals are refractory to vaccination, likely due to the SIE phenomenon. Improvement of vaccination strategies and diagnosis of subclinical forms of CSF is imperative for CSF eradication.

**Electronic supplementary material:**

The online version of this article (10.1186/s12917-019-1982-x) contains supplementary material, which is available to authorized users.

## Background

Classical swine fever (CSF) is one of the most devastating diseases in the pig industry worldwide, affecting domestic pigs and wild boars. This disease is endemic in Asia, Central and South America, and some Eastern European countries [1]. CSF virus (CSFV), the aetiological agent, belongs to the Pestivirus genus of the Flaviviridae family [2]. Three degrees of virulence (low, medium and high) have been described for CSFV strains, which have also been linked to different clinical manifestations of the disease [3]. Pigs infected with low-virulence strains may develop chronic CSF and can shed the virus continuously or intermittently for months, representing a constant source of reinfection [3]. Additionally, the role of low-virulence strains in “pregnant carrier sow syndrome”, which can lead to congenital infection of the foetus by trans-placental transmission, has been known for more than 50 years [4–7].

Modified live attenuated vaccines based on the C-strain have played a relevant role in the implementation of eradication programmes for CSF [8–12]. These vaccines present several advantages that facilitate their use in developing countries, inducing high titres of neutralizing antibodies that provide protection against highly virulent CSFV strains [9, 13]. Moreover, this type of vaccine confers protection against CSFV even in the absence of neutralizing antibodies a few days post vaccination (dpv) [8, 9, 13, 14]. In addition to horizontal protection, modified live attenuated vaccines based on the C-strain also induce protection in pregnant sows as early as 5 dpv, preventing viral trans-placental transmission [8, 9]. Although the immune response induced by this type of vaccine does not allow differentiation of infected animals from vaccinated animals (the DIVA concept) [8, 9, 12], this vaccine continues to be used in endemic countries (a majority of which are developing countries). The reasons underlying the worldwide distribution of this vaccine are its high efficacy and safety as well as its relatively low cost of production [10].

It has been previously discussed that CSF has not been controlled despite long-term vaccination programmes with live attenuated C-strain vaccines conducted in endemic areas [11, 15, 16]. Factors associated with variability, quality, and stability of the vaccine batches produced; loss of the cold chain; and lack of vaccination coverage, mainly in rural areas with poor accessibility and backyard farms, among others, have been used to explain this discrepancy [9, 11, 15, 16].

Recent studies have demonstrated evolutionary capacity of CSFV in endemic conditions under inefficient vaccination programmes. In this regard, it has been hypothesized that circulation of new viral variants when the immunity induced by the vaccine is not sterilizing might favour viral persistence in the swine population [11, 15, 17, 18]. Long-term endemism of CSF is characterized by the circulation of moderate- and low-virulence strains [1, 11]. Therefore, laboratory support for accurate CSFV diagnosis is fundamental for efficient disease control. Unfortunately, not all endemic countries have the necessary resources to achieve this goal, and only clinical diagnosis is performed. Hence, the lack of laboratory diagnostics may also explain the complex epidemiological scenario in some endemic countries.

In addition to the persistent viral infection generated after trans-placental transmission, previous works have reported that CSFV is also able to generate persistent postnatal infection in both domestic pigs and wild boars [19, 20]. Subsequently, it was shown that pigs with persistent postnatal infection were unable to elicit a specific immune response to a CSFV live attenuated vaccine, with viral vaccine RNA undetectable in these pigs after vaccination via the superinfection exclusion (SIE) mechanism [21, 22]. These studies were performed under experimental conditions, with one of the few scientific reports showing the possible negative impact that persistently infected animals may have on vaccination programmes, which could complicate CSF control in endemic countries.

Given this background, the present study aimed to determine whether subclinical forms of CSF, which may be misdiagnosed in the field, are present at the time of vaccination in a conventional farm from an endemic country. Likewise, this study aimed to evaluate vaccine efficacy for these types of infections in field conditions, an important aspect that is poorly understood to date.

## Results

### CSFV detection and antibody response in sows

Despite being vaccinated, the rectal swabs of five of the sows were CSFV RNA positive (from B to F), with Ct values corresponding to high (litter C) to moderate (litters B and D) and low (litters E and F) viral RNA loads. In contrast, the sow from litter A was negative. The results obtained from tonsil samples corresponded with those from rectal swabs, i.e., there were five positive sows (from B to F) and one negative sow (sow A). The sow from litter F showed a Ct value less than 28, and the others showed values greater than 29. Notably, although the rectal swabs of five sows were positive, the sera of only two of these sows were positive, showing low viral RNA loads (sows B and C had Ct values of 30 and 32, respectively).

In terms of humoral response, notably, only the sow from litter E showed an antibody response (55.3% blocking) in the ELISA test. With the exception of the animals from the six litters under study, all the animals from the farm were slaughtered once the samples collected at weaning were found to be CSFV RNA positive, and strict biosecurity measures were applied in the farm.

### CSFV detection and antibody response in piglets at weaning

At the day of weaning (33 days after birth), the rectal swabs of all piglets in four of the six litters were CSFV RNA positive (litters B, C, D and F) and showed Ct values corresponding to high (Ct value less than 23) and moderate (Ct values between 23 and 28) viral RNA loads (Table 1). In the other two litters, only two out of nine animals were CSFV RNA positive with high RNA viral loads in rectal swabs in litter A, whereas in litter E, eight out of eleven were CSFV RNA positive with high and moderate RNA viral load in rectal swabs (Table 1). Likewise, the results obtained for sera were similar to those obtained for rectal swabs. All serum samples from litters B, C, D and F were CSFV RNA positive, showing Ct values corresponding with high viral RNA loads (Ct value less than 23) in 38 out of 42 piglets (Table 1). Different profiles were observed in the two remaining litters. In litter E, five out of eleven piglets were CSFV RNA positive, with two animals exhibiting low viral RNA loads, one with a moderate RNA load and the other two with high viral RNA loads (Table 1). In litter A, the serum samples of four out of nine animals were positive (44.4%), three with Ct values corresponding to low RNA loads and one with a moderate viral RNA load. In general, the subclinical infection detected in the piglets from the present study at weaning included 82% animals that were viraemic. Among the remaining 18%, most of the piglets, although not viraemic, were positive in rectal swabs.

**Table 1:**
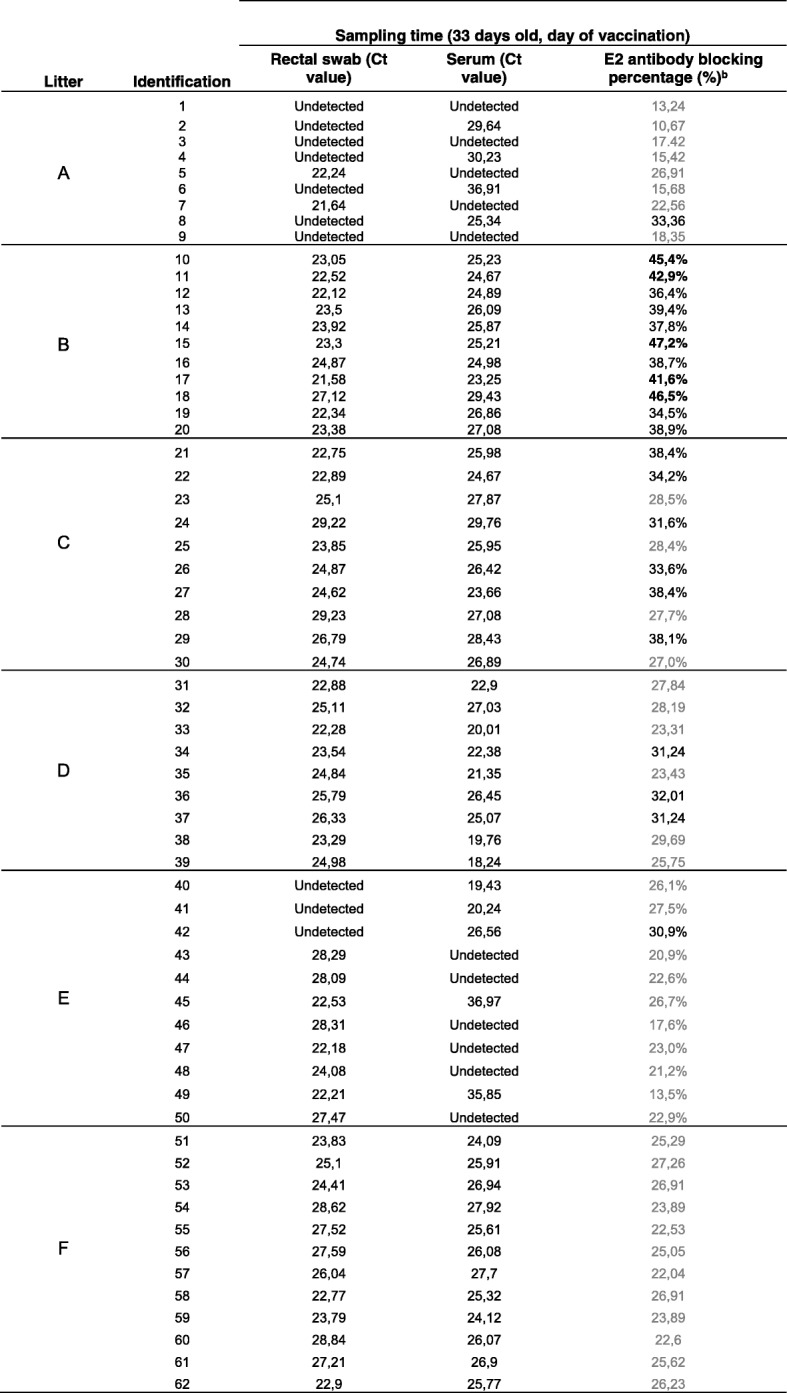
Ct values from rectal swabs and sera obtained by CSFV-specific qRT-PCR and CSFV-specific antibodies at weaning

^b^The antibody test result against the E2 glycoprotein was considered positive when the blocking % was more than 40% (in bold). Values between 30 and 40% were considered doubtful (in black), and values less than 30% were considered negative (in grey)

In terms of humoral response, only five out of eleven piglets from litter B showed a specific antibody response to E2 glycoprotein, as detected by ELISA, whereas the remaining piglets from the study were negative. Regarding the ELISA-positive samples, the antibody response levels measured as blocking percentage values were between 42 and 47% (Table 1). These results were confirmed by the NPLA, resulting in low neutralizing antibody titres of 1:25 in the five sera.

### Clinical manifestations registered at weaning and after vaccination

Notably, at weaning, all the animals were apparently clinically healthy (Table 2), exhibiting a weight gain similar to the normal weight gain observed in Cuba in this type of commercial farm (data not shown); likewise, the absence of clinical symptoms was correlated with the absence of mortality at weaning. At 21 dpv, clinical signs were registered in only litter E, such as anorexia, conjunctivitis, weakness of hindquarters, dyspnoea, diarrhoea, incoordination of movements and nervous disorders; three animals died suddenly, and the remaining animals were euthanized at 21 dpv. Lesions found after necropsy included petechial haemorrhages in the kidney, bladder and stomach; marginal infarcts of the spleen; haemorrhages; and necrosis in the tonsils (Table 2). In the remaining litters, there were no obvious clinical signs; two cases of sudden death occurred in litters A and D, while cases of animals with polyarthritis were recorded in litters B and C. A mortality rate of 8% was obtained between 21 and 96 dpv, with a 14% mortality observed during the entire study. Additionally, the mortality for each litter was also determined (Table 2). Interestingly, in litters B, C and F, no anatomopathological lesions in internal organs were observed after necropsy. Finally, the three litters that remained until 96 dpv showed growth retardation from approximately 36 dpv until the end of the study (litters A, D and F), with clear signs of apathy, anorexia, diarrhoea, dyspnoea and conjunctivitis observed at that time in some animals. The largest accumulation of anatomopathological lesions of haemorrhages in tonsils and kidneys, enteritis and even development of interstitial pneumonia were observed in litters A and D, which remained in the study until 96 dpv; however, the mortality percentage in both litters was 22.2% (Table 2).

**Table 2:**
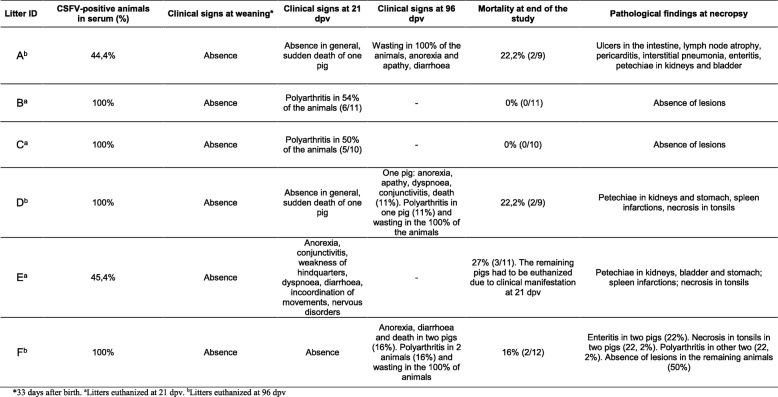
CSFV-positive animals at weaning and clinical signs, mortality rates and pathological findings in the litters under study

### CSFV detection in piglets after vaccination

The presence of CSFV RNA in sera, rectal swabs and tonsils was monitored by qRT-PCR at different times post vaccination. After 21 dpv, 98% of the piglets from the six litters were unable to clear the virus, with a majority of the piglets exhibiting high and moderate viral RNA loads in sera and rectal swabs (Tables 3 and 4). At 21 dpv, serum samples from the six litters that were positive by qRT-PCR with Ct values below 28 were verified by a viral isolation test in PK-15 cells.

**Table 3:**
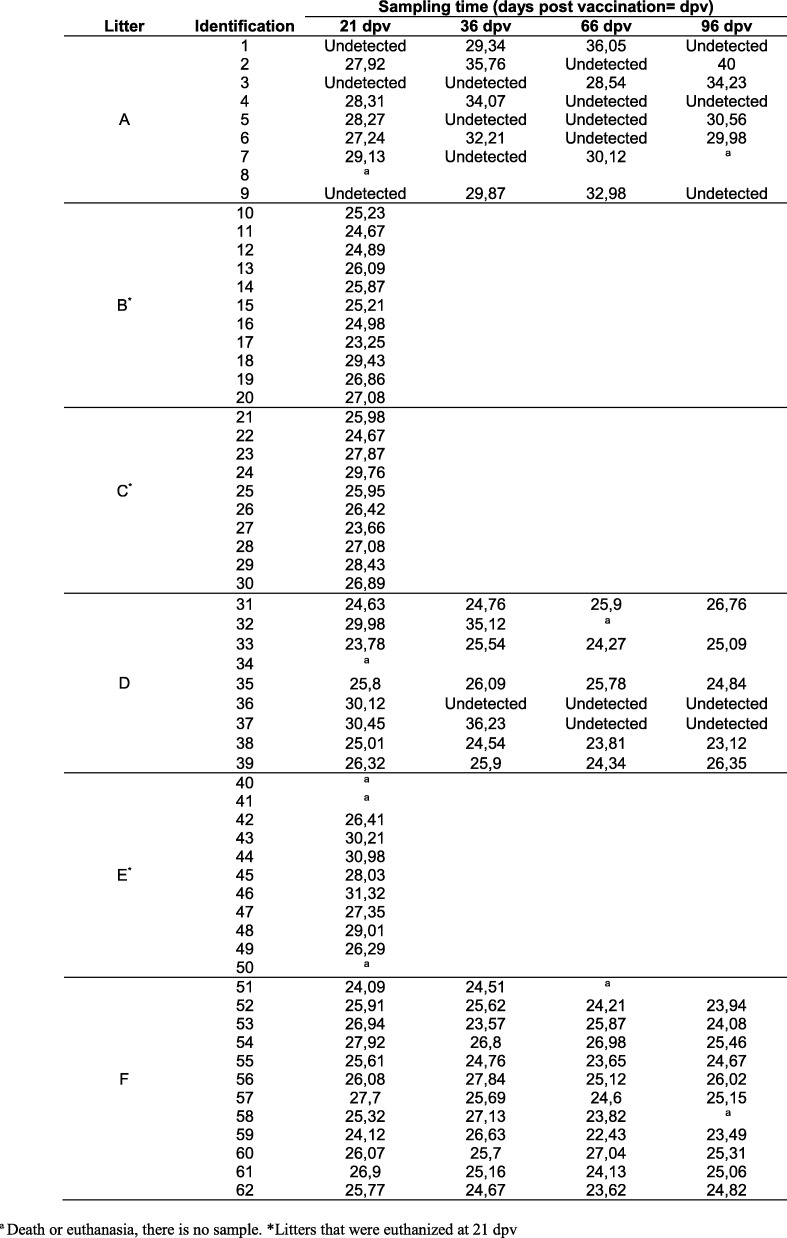
Ct values in rectal swabs obtained by specific qRT-PCR at different times post vaccination

**Table 4:**
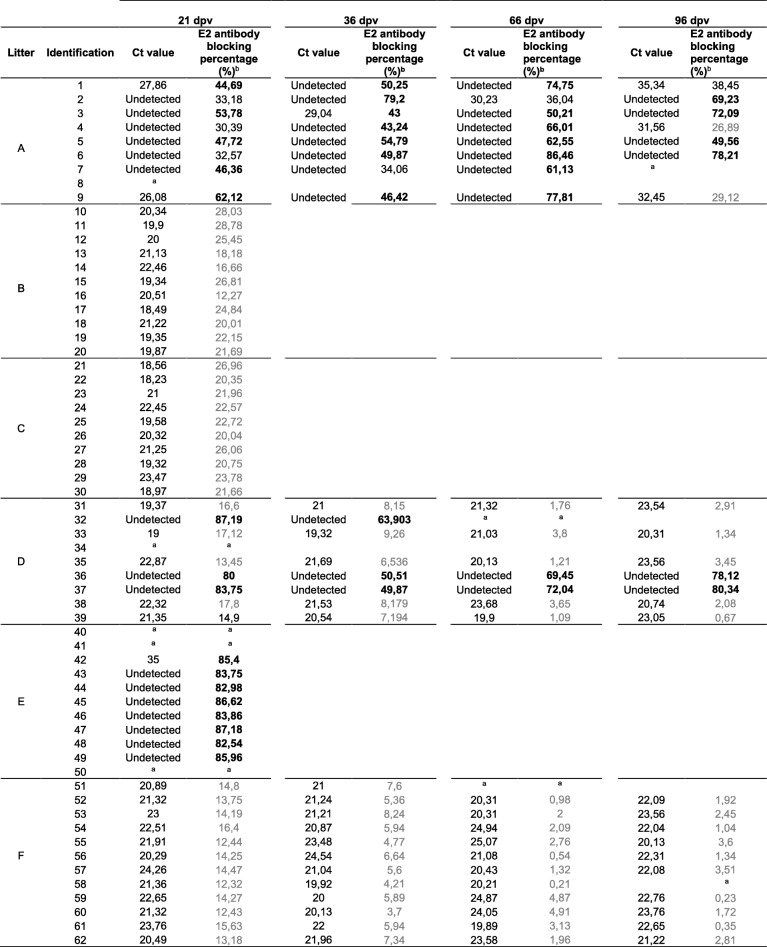
CSFV RNA detection in sera and CSFV-specific antibodies after vaccination

^a^Death or euthanasia, there is no sample. ^b^The antibody test result against the E2 glycoprotein was considered positive when the blocking % was more than 40% (in bold). Values between 30 and 40% were considered inconclusive (in black), and values less than 30% were considered negative (in grey)

**Fig. 1:**
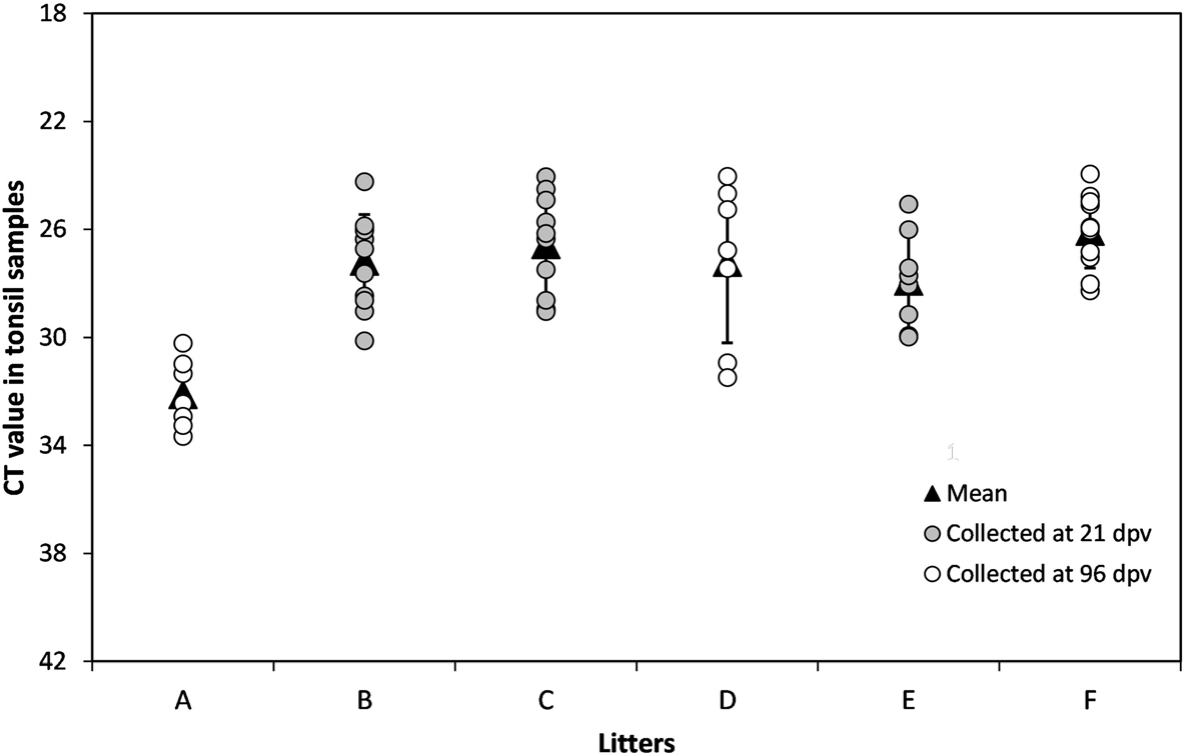
CSFV RNA detection in tonsil samples from piglets. CSFV viral RNA was detected using qRT-PCR [23] in litters either at 21 dpv (grey dots) or 96 dpv (white dots). Viral RNA load is expressed as the Ct value; the mean Ct value for each litter is also indicated (black triangle). Ct values equal to or less than 42 were considered to indicate positive results

Notably, piglets from litters B, C and F showed the highest viral RNA loads during the study, with strong viral RNA signals observed in sera, rectal swabs and tonsils (Tables 3, 4 and Fig. 1), and showed constant and similar Ct values during the study. In the remaining litters (A, D and E), the viral RNA load ranged from low to moderate and was undetected in some cases. In these litters, rectal swab samples allowed the detection of a greater number of positive animals than serum samples (Tables 3, 4 and Fig. 1). In particular, a majority of the pigs (seven out of nine) in litter A tested negative for viral RNA in serum samples at 21 dpv. After this time, at 33, 66 and 96 dpv, positive samples were detected intermittently, although with low viral RNA loads. Nevertheless, during the period from 21 to 96 dpv, the rectal swabs from all animals were positive at some point (Tables 3 and 4). Finally, in pigs from litter D, two different profiles were detected; three of these pigs developed some antibody response to the virus, as two animals were able to clear the virus in sera and rectal swabs from 33 to 96 dpv. In the remainder of the pigs, constant viraemia was observed during the study until 96 dpv with a high viral RNA load. Furthermore, the tonsils of the animals from this litter were also positive, with a low viral RNA load observed in the two animals that cleared the virus in sera. In contrast, animals that showed persistent viremia exhibited moderate and high viral RNA loads (Tables 3, 4 and Fig. 1). Additionally, despite the intermittent viral RNA detection during the study in litter A (Tables 3 and 4), the tonsils of all the animals were positive, although with low viral RNA loads. Finally, the tonsils of all the animals under study were positive at either 21 dpv or 96 dpv (Fig. 1).

### Antibody response profile detected after vaccination

**Fig. 2:**
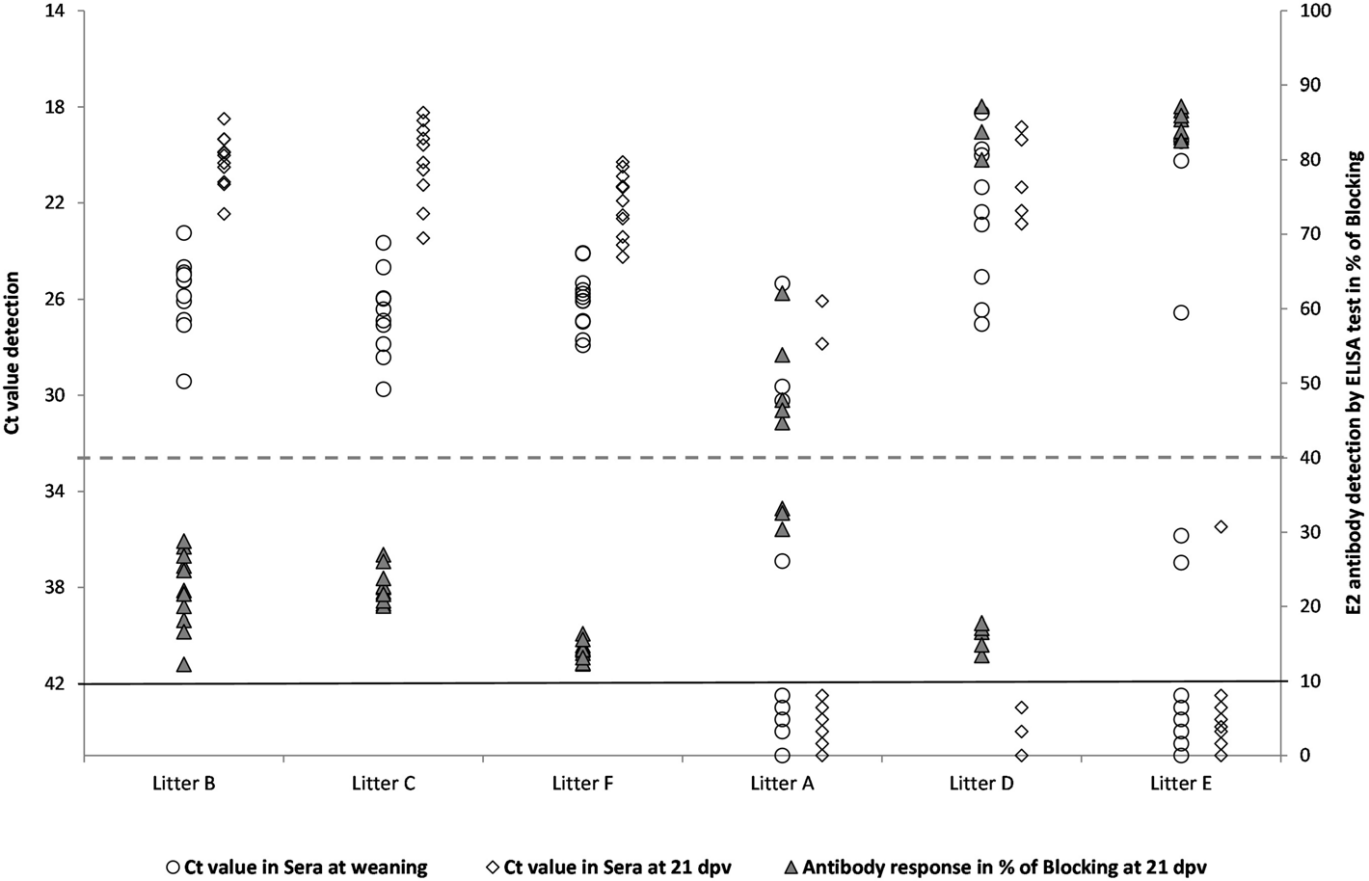
CSFV RNA detection in serum samples at weaning and 21 dpv versus antibody response after vaccination. CSFV RNA loads in sera expressed as Ct values (left Y axis) at weaning (white dots) and 21 dpv (white rhombuses). Ct values equal to or less than 42 were considered to indicate positive results (black line). Antibody response against the E2 glycoprotein detected by a commercial ELISA test (IDEXX) expressed as blocking % (right Y axis); samples with blocking % values more than 40 were considered positive (dotted line)

**Fig. 3:**
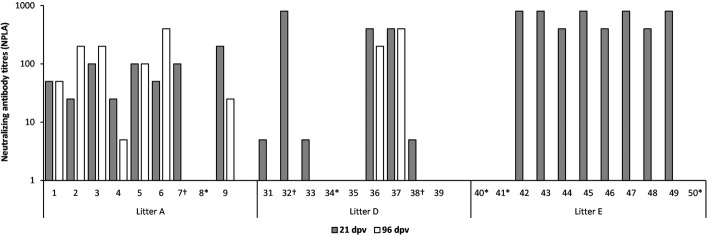
Neutralizing antibody response in piglets. Neutralizing antibody titres were detected by NPLA at 21 dpv (grey bars) and 96 dpv (white bars). Only the litters in which neutralizing antibody titres were detected are shown. * indicates animals that died before 21 dpv. † indicates animals that died between 21 and 96 dpv

Notably, none of the animals in litters B, C and F showed an antibody response detected by ELISA at 21 dpv (litters B and C) or 96 dpv (litter F). Among the remaining three litters, in litter A, five out of nine piglets were able to seroconvert at 21 dpv (Table 4 and Fig. 2). Furthermore, between 33 and 96 dpv, intermittent detection of antibodies in animals from this litter was registered. In terms of neutralizing antibody titres, only four pigs were capable of clearing the virus in sera, as determined by the NPLA at 96 dpv, with neutralizing antibody titres from 1:100 to 1:400 (Fig. 3).

In the case of litter D, only three pigs showed CSFV-specific antibodies to E2 glycoprotein and neutralizing antibody titres at 21 dpv (Table 4 and Fig. 3). One of these pigs died (pig number 32) despite being the animal with the highest level of neutralizing antibodies generated against infection. The other two antibody-positive pigs showed neutralizing antibody titres ranging from 1:200 to 1:400 at 96 dpv (Fig. 3).

Finally, in litter E, five pigs showed CSFV-specific antibody responses, as measured by ELISA and the NPLA (Figs. 2 and 3). This group generated the highest neutralizing antibody response at 21 dpv, with titres ranging from 1:400 to 1:800 (Fig. 3).

### E2 sequence determination for each litter and sequence analysis

The complete E2 sequences from tonsil samples of one animal from each litter studied were determined. Notably, the six consensus sequences obtained were identical and were deposited in the European Nucleotide Archive under the accession number LT985811. This viral strain was isolated and designated Pinar del Rio/2016. The full-length E2 gene of Pinar del Rio/2016 (LT985811), as well as all the available full-length E2 gene sequences from Cuban CSFV strains, were analysed as described in Methods section. By in silico analysis, the amino acid at position 72 (R) on the E2 glycoprotein was identified under positive selection pressure for the strain LT985811 with a 0.01 level of significance (2LnL = 12.6 M2vsM1 and 2LnL = 36.8 M8vsM7). Likewise, sites 20 (L) and 195 (N) were also identified in silico as positions with adaptive advantage in the above-mentioned virus with a 0.01 significance level (θLTR = 28.85684). Other mutations without statistical significance were detected in the CSFV strain from the present study, namely, 3AxV and 7DxG in the external loop of the B/C domain, 91IxV in the A/D domain and 210DxN and 228AxV in the transmembrane domain (Additional file 1: Figure S1).

## Discussion

In the present work, the coexistence of two forms of subclinical CSF was demonstrated: (i) chronic CSF and (ii) persistent CSF, likely transmitted congenitally from sows to their litters. Nevertheless, a postnatal persistent infection in some of these animals cannot be ruled out considering the ability of CSFV to generate this form of infection soon after birth [20]. Although both forms of infection can be considered as chronic CSF manifestations, since both exceed 28 days of infection [1, 24], the knowledge and distinction of the pathogenesis of both types of infections is relevant for accurate diagnosis and control in endemic areas. One of the main differences between pigs with persistent CSFV infection and pigs suffering the chronic form is that, in chronic infection, a specific immune response against CSFV can be generated, although inefficiently, and in some cases, the antibody response can mask the detection of the virus in sera [20, 25]. Considering this fact, even though antibodies were intermittently detected in some animals, the non-viraemic animals detected in this study that exhibited positive rectal swabs and tonsils were considered to be chronically infected, such as piglets from litters A, D and E.

In contrast, persistently infected animals have been previously defined as CSF-infected pigs that show permanent viremia with high viral titres over long periods of time and are unable to generate a specific immune response to the virus, mainly in terms of the humoral immune response [20, 25]. Recently, a type I and II interferon response blockade has been demonstrated in animals with persistent CSFV infection [20, 22]. This phenomenon explains the immunological anergy in pigs with persistent CSFV infection, blocking the immunological mechanisms of the host to fight the infection [20, 26]. Notably, CSFV persistent form was the predominant infection detected in the animals from the present study, including those from litters B, C and F and some piglets from litter D.

Although other porcine pathogens were not diagnosed in this study, previous work has shown that, in Cuba, CSFV is circulating in concomitance with other viral porcine pathogens, such as porcine circovirus 2 (PCV-2) and porcine parvovirus (PPV) [27]. However, considering the high relevance, future studies will be carried out to understand the implications of other porcine pathogens involved in coinfection with CSFV circulating in endemic scenarios. Unfortunately, currently, the resources have been used to detect the presence of subclinical forms of CSF, given the high impact of CSFV on the porcine immune system and given that CSF is considered a notifiable disease by the World Organisation for Animal Health (OIE).

Previous results showed that chronic forms of CSF can be fatal, with clinical manifestations similar to those described in the present study, as was the case for piglets from litter E [1, 24] (Table 2). This effect is probably due to the immunosuppression caused by persistent CSFV infections [20]. Previous work performed under laboratory conditions has indicated that pigs that exhibit CSFV permanent viremia for more than 30 days post infection may develop a similar clinical condition [20, 21]. Our data also show the clinical evolution over time of animals with persistent CSFV infection. In these animals, signs of growth retardation between 21 and 36 dpv were observed. It was not until after 66 dpv (approximately 3 months) that, in a sporadic manner, some of the animals developed severe symptoms, as previously described for the chronic form of CSF [24]. However, animals showed permanent viremia until the end of the study (96 dpv; more than 4 months of infection), exhibiting the inability to seroconvert. Likewise, given that the animals in our study belonged to a commercial farm, different aetiological agents could be associated with the clinical signs registered in some of these animals, which supports the importance of identification of concomitant infections that may be related, as described above; these infections will be identified in future studies.

Previous work has shown detection of a low RNA load of the lapinized C-strain vaccine by specific qRT-PCR [10] in serum and rectal swab samples soon after vaccination in Pestivirus-free vaccinated pigs [21]. Based on the levels of CSFV RNA loads detected in the rectal swabs and tonsils from vaccinated sows, as well as the inconsistent humoral response observed after vaccination in these animals, we determined that five of these animals were asymptomatic CSFV carriers (sows from B to F). Only one vaccinated sow (sow E) was antibody positive at weaning, although at very low levels. The results of the present study indicate that antibody response detection and CSFV diagnosis in serum samples from subclinically infected CSFV animals may be hindered by the coexistence of some antibodies and CSFV [20]. Therefore, in addition to detecting the virus in tonsils or tonsil scrapings, these results indicate the effectiveness of using samples such as rectal swabs for the detection of subclinical forms of CSF.

The failure in the response to vaccination in the sows was suggested by the lack of maternal immune response to protect piglets from either prenatal or postnatal infections. Factors associated with poor handling and malpractice in vaccination have been widely discussed to explain the constant failures in vaccination in endemic areas [11, 15, 16]. Thus, these results prove how vaccine failure in the field may allow viral persistence in an endemic scenario. Improvement in vaccine quality, as well as optimization of vaccination dose and schedule, in gilts and sows may aid the prevention of CSFV carrier generation [28]. Nevertheless, the reason underlying the lack of effective maternal immunity in sows after vaccination must be studied in depth.

Recently, SIE or homologous interference has been described as a mechanism that can interfere with CSFV vaccine efficacy in pigs with persistent postnatal CSFV infection under experimental conditions [21, 22]. Here, we show that this mechanism may also occur in animals suffering from subclinical CSF, either chronic or persistent, remaining unnoticed at the time of vaccination in field conditions. Pigs with persistent infection from the present study were unable to seroconvert at any time during more than 4 months under study. Likewise, vaccination was also ineffective in litters suffering from a chronic form of CSF. Therefore, vaccination against subclinical forms of CSFV is counterproductive and generates additional expenses for control programmes. Thus, along with improvement in the vaccination strategy, efforts must also be made in the diagnosis of these CSF disease forms.

With respect to the nature of the circulating viral strain, notably, the consensus sequence identified in the full-length E2 gene of the virus recovered from the six litters was the same regardless of the different CSF forms recorded. Therefore, our results corroborate, in a field infection, previous results showing CSFV stability [11, 29]. The CSFV Pinar del Rio strain isolated in 2010 (Pinar del Rio/2010) was phylogenetically the closest relative to the Pinar del Rio/2016 strain recovered in the present study. Previous studies have determined that the full-length E2 sequence is the most reliable sequence for phylogenetic analysis of CSFV [17, 30]. Position 72 (R) on the E2 protein, previously described in Pinar del Rio/2010 as a positive selection site for Cuban strains [15], was also identified in the Pinar del Rio/2016 isolate. This finding may suggest the relevance of this codon in the evolution of the CSFV 1.4 genotype in the Cuban endemic scenario. Likewise, codon 20 (L), previously described as a position associated with adaptive advantage in the CSFV Pinar del Rio/2010 strain [17], was also found in the strain from the present study. Moreover, codon 195 (N), present in only the CSFV Pinar del Rio/2016 strain, was also identified in silico as a position associated with adaptive advantage. Further studies will clarify the implications of these findings in the evolution and pathogenesis of CSFV.

## Conclusion

The presence of chronic and persistent CSF infections was demonstrated in an endemic scenario under vaccination. The CSF persistent infection was the predominant form in the animals studied. Here, we provide one of the few reports showing that, in field conditions, subclinical infections in animals are not detected by clinical diagnosis and, despite being infected with CSFV, the animals are vaccinated, rather than diagnosed and eliminated. Animals with persistent and chronic CSFV infections are refractory to vaccination, likely due to the SIE phenomenon. Hence, CSFV eradication in some endemic areas will require the elimination of persistently and chronically infected animals by relying heavily on laboratory tools for effective diagnosis. Likewise, CSF control programmes may include a vaccination strategy that confers sterilizing immunity to avoid the generation of subclinical infections in the field. Therefore, government support and financial aid will be key factors to ensure the institution of policies for effective control and eradication of this disease globally.

## Methods

### Cells and viruses

The porcine kidney cell line PK-15 (ATCC CCL 33, U.S.A.) was cultured in pestivirus-free Dulbecco’s modified Eagle medium (DMEM; Lonza, Switzerland) supplemented with 5% foetal bovine serum (FBS) (Euroclone, Italy) at 37 °C in 5% CO2. The cell monolayer was incubated with 100 μl of serum. Following 1 h of adsorption, the serum was removed, and the cells were cultured in DMEM supplemented with 2% FBS. After 48 h of incubation, a peroxidase-linked assay (PLA) [31] was used for viral identification.

A lapinized live attenuated C-strain-based vaccine (Labiofam strain) belonging to subgenotype 1.1 (batch number 16001) and used in Cuba since 1965 for vaccination against CSF [15, 32] was employed in this study. This vaccine batch had been previously tested in 33-day-old naïve pigs, and all the pigs showed a specific CSFV antibody response at 21 dpv, as measured by ELISA (IDEXX, Netherlands).

### Experimental design

This study was performed in a recently repopulated Cuban commercial pig farm located in the western region. Six randomly chosen sows and their respective litters (designated A to F) were included. The first-parity sows had been vaccinated with one dose of the lapinized CSFV C-strain vaccine (Labiofam strain) at weaning and again at 6 months before delivery. The experiment was initiated at weaning (33 days after birth), at which time, vaccination of piglets from the farm was carried out. At this time, piglets were identified individually (1 to 62), and the distribution among litters was as follows: litter A (piglets 1 to 9); B (10 to 20); C (21 to 30); D (31 to 39); E (40 to 50); F (51 to 62). After identification, sera and rectal swabs were collected from the litters and the corresponding sows. The sows were euthanized, and tonsil samples were collected. Subsequently, the piglets were vaccinated using a pig dose, equivalent to 100 protective doses (PDs), of a lapinized live attenuated C-strain-based vaccine (Labiofam strain) by intramuscular injection in the neck. At 21 dpv, sera and rectal swabs were obtained from the 62 piglets. The piglets from litters B, C and E were then euthanized, and tonsils were collected. Sera and rectal swabs were obtained from the animals from the remaining litters (A, D and F) at 36, 66 and 96 dpv (end of experiment), and tonsil samples were obtained. Additionally, clinical signs were registered by the veterinarian from the farm.

All pigs were handled according to the rules described in the manual of good practices for porcine production from the Cuban Republic [33]. International standards for animal welfare were used following the regulations of the Animal Health Directory (DSA). The Ethics Committee of the Cuban Ministry of Agriculture (MINAGRI) approved the study, and all efforts were made to minimize animal suffering. Animals were euthanized by bleeding by inserting an appropriately sized knife into major artery following stunning, according to the current protocol described in the OIE Terrestrial Animal Health Code [34].

According to the rules for the CSF control programme in the country, in case of positive CSFV detection, only the animals under study were to be maintained following strict biosecurity measures, and the remaining animals from the farm were to be eliminated.

### Detection of CSFV RNA and virus isolation

RNA was extracted from all of the samples using the RNeasy Mini Kit (Qiagen, Netherlands) according to the manufacturer’s instructions. The presence of CSFV RNA in sera, rectal swabs and tonsil samples was analysed by qRT-PCR [23]. Threshold cycle values (Ct) equal to or less than 42 were considered to indicate positive results. Samples in which fluorescence was undetectable were considered negative. Ct values below 23 were considered high, values from 23 to 28 were considered moderate, and values from 29 to 42 were considered low RNA viral loads [35]. Serum samples that were positive by qRT-PCR at 21 dpv were tested by virus isolation in PK15 cells, as described above.

### E2-specific and neutralizing antibody detection

Piglet serum samples were tested with a neutralization PLA (NPLA) at weaning (33 days old, 0 dpv) and at 21 and 96 dpv [36]. The titres were expressed as the reciprocal dilution of serum that neutralized 100 TCID50 of the Margarita strain in 50% of the culture replicates. Detection of E2-specific antibodies was performed using a commercial ELISA kit (IDEXX, Netherlands) following the manufacturer’s recommendations in serum samples collected at weaning from sows and piglets. In addition, sera from piglets were also evaluated at 21, 36, 66 and 96 dpv. Samples with blocking percentage values below 30% were considered negative, those with values between 30 and 40% were considered doubtful and those with values ≥40% were considered positive.

### Full-length E2 encoding sequence determination

The sequence of the full-length E2 region was determined from tonsils obtained from one piglet of each litter as previously described [30]. The one-step RT-PCR protocol was performed using the commercially available One-Step RT-PCR Kit (Qiagen, Netherlands). The sequencing reactions were conducted under the BigDye™ terminator-cycling conditions (Thermo Fisher Scientific, U.S.A.) using an ABI 3130 × L instrument. Forward and reverse sequences obtained from each amplicon were assembled using the Contig Express application in Vector NTI software, version 11 (Invitrogen, U.S.A.).

### Sequence analysis

Multiple sequence alignment was conducted for all available full-length E2 gene sequences from Cuban strains (CSFV/Margarita/1958 [JX028201, AJ704817], CSFV/Santiago de Cuba/2011 [JX028203], CSFV/Holguin/2009[JX028202], CSFV/Pinar del Rio/2010 [JX028204, KX576461], and the new sequences obtained in the present study). Multiple sequence alignment was performed using the algorithm Clustal W (EMBL-EBI, United Kingdom) included in the program BioEdit Sequence Alignment Editor [37]. Positive selection analysis from the complete E2 gene was carried out using several models (M0, M1, M2, M7 and M8) available in the CODEML module of the PAML 4.7 software package [38]. Finally, functional divergence among the Cuban sequences was assessed by estimation of the coefficients of type-I functional divergence (θI) using DIVERGE 3.0 software [39]. The 3D structure and surface of the E2 protein was constructed as previously described [17] for the Cuban ancestral strain “Margarita/1958” [AJ704817] [11], the previously described “Pinar del Rio/2010” [KX576461] strain [11] and the new strain obtained in the present study.

## Additional file

Additional file 1: **Figure S1.** Representation of the mutation pattern for Cuban CSFV strains on the 3D structure of the predicted model of the E2 protein of CSFV. The 3D structure and surface of the Cuban ancestral strain “Margarita/1958” [AJ704817], the previously described “Pinar del Rio/2010” [KX576461] strain [11] and the new strain “Pinar del Rio/2016”[LT985811] are presented. The in silico mutations associated with positive selection (red), associated with adaptation (blue) and not linked previously with any evolutionary advantage (yellow) are denoted. The antigenic region B/C (gold), antigenic region A/D (ruby red) and transmembrane domain (grey) are located on monomer B. (JPG 94 kb)

## Data Availability

The datasets used and/or analysed in the present study are available from the corresponding author on reasonable request.

## Abbreviations

CSF: Classical swine fever
CSFV: Classical swine fever virus
Ct: Threshold cycle
DIVA: Differentiating infected from vaccinated animals
DMEM: Dulbecco’s modified Eagle medium
dpv: Days post vaccination
DSA: Animal Health Directory
FBS: Foetal bovine serum
MINAGRI: Ministry of Agriculture
NPLA: Neutralization peroxidase-linked assay
PCV-2: Porcine circovirus 2
PD: Protective dose
PK-15 cells: Porcine kidney-15 cell line
PLA: Peroxidase-linked assay
PPV: Porcine parvovirus
qRT-PCR: Quantitative reverse transcription polymerase chain reaction
RNA: Ribonucleic acid
TCID_50_: 50% tissue culture infective dose
