# Supplementary figures and images for: Investigation of chronic and persistent classical swine fever infections under field conditions and their impact on vaccine efficacy
Source: BMC Vet Res. 2019 Jul 15;15:247. doi: 10.1186/s12917-019-1982-x (PMC6632193; doi:10.1186/s12917-019-1982-x)

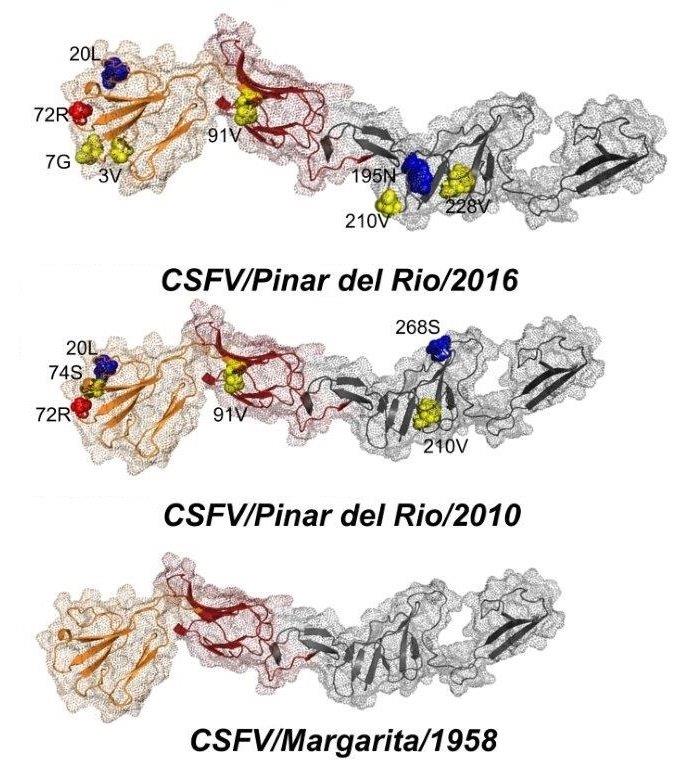

Supplement: Supplementary file 1 — Figure S1. Representation of the mutation pattern for Cuban CSFV strains on the 3D structure of the predicted model of the E2 protein of CSFV. The 3D structure and surface of the Cuban ancestral strain “Margarita/1958” [AJ704817], the previously described “Pinar del Rio/2010” [KX576461] strain [11] and the new strain “Pinar del Rio/2016”[LT985811] are presented. The in silico mutations associated with positive selection (red), associated with adaptation (blue) and not linked previously with any evolutionary advantage (yellow) are denoted. The antigenic region B/C (gold), antigenic region A/D (ruby red) and transmembrane domain (grey) are located on monomer B. (JPG 94 kb) [file 12917_2019_1982_MOESM1_ESM.jpg]
